# Supplementary material for: Sex-Based Clinical Outcome in Advanced NSCLC Patients Undergoing PD-1/PD-L1 Inhibitor Therapy—A Retrospective Bi-Centric Cohort Study
Source: Cancers (Basel). 2021 Dec 24;14(1):93. doi: 10.3390/cancers14010093 (PMC8750380; doi:10.3390/cancers14010093)
Supplement: Supplementary file 1 [file cancers-14-00093-s001.zip › cancers-1498830-supplementary.pdf]

<u>**Supplementary analysis S1.**</u> Statistical approach to the impact of numeric differences between men and women on progression-free and overall survival (PFS/OS)

Problem: The ratio between male and female patients is unequal, which may have influenced PFS and OS.

Question: Would PFS and OS change, if the ratio was equal?

Results as presented in the original analysis:

cohort	analysis	n	n female	Median (95% CI) female vs. male	*p*-value (log rank)
ICI monotherapy	PFS	228	92 (40.4%)	3M (3-6) vs. 3M (3-5)	0.273
	OS	228	92 (40.4%)	10M (6-14) vs. 10M (8-14)	0.592
ICI-chemotherapy	PFS	80	33 (41.3%)	5M (3-NA) vs. 6M (5-10)	0.780
	OS	80	33 (41.3%)	10M (7-NA) vs. 15M (10-NA)	0.398

ICI monotherapy cohort (n = 228)

PFS

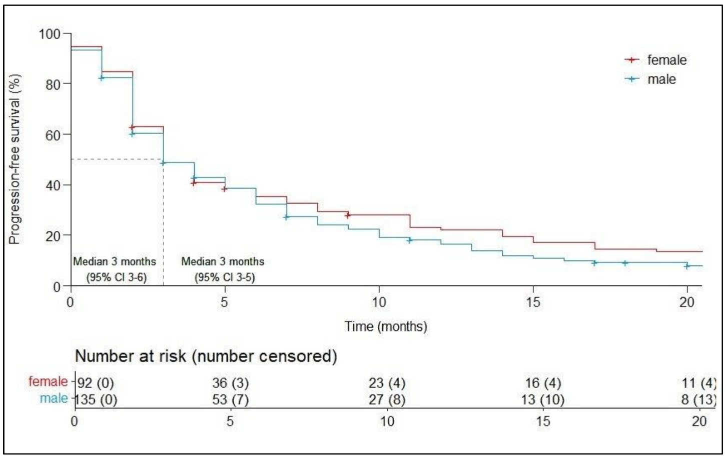

OS

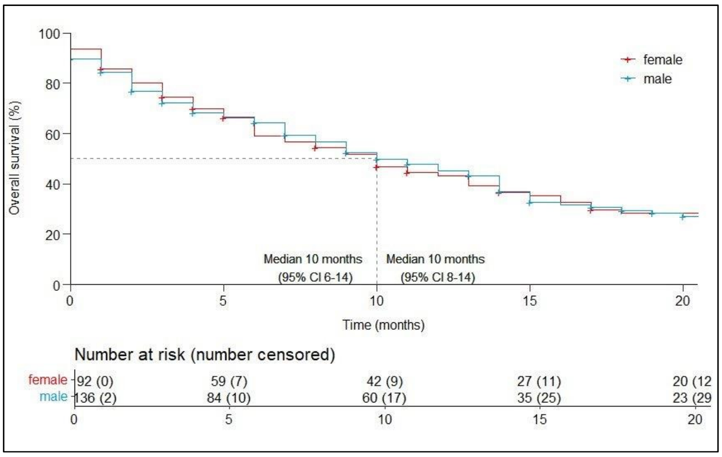

ICI-chemotherapy cohort (n = 80)

PFS

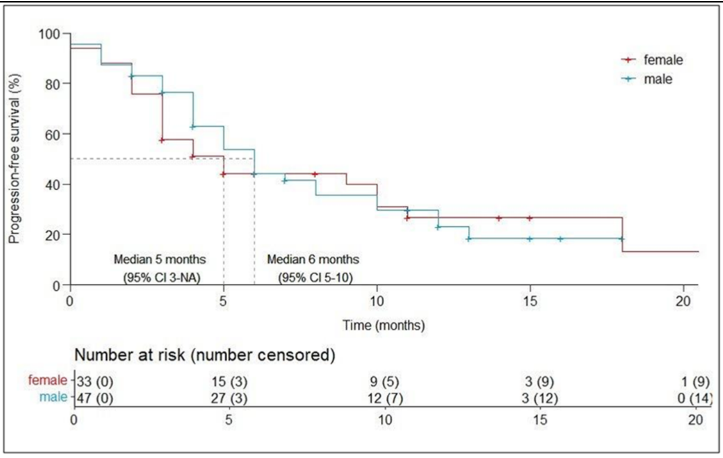

OS

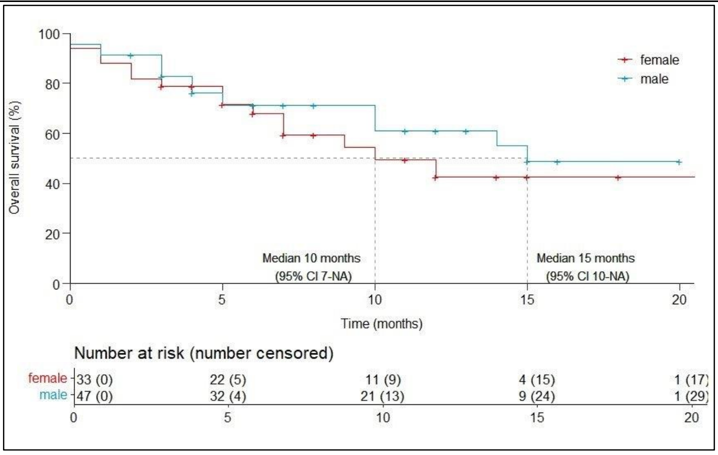

Solution: Extension of the current female patient datasets with simulated data.

Methods: The aim is to derive cohorts with a balanced male-to-female-ratio. Therefore, the male population is fixed in both datasets, and based on the key characteristics of the existing female patients, additional female cases are simulated using the "survsim" package in R (R: A Language and Environment for Statistical Computing; Version 3.6.0; https://www.R-project.org). The simulated cases are added to the original datasets:

ICI-monotherapy cohort: 44 female subjects

ICI-chemotherapy cohort: 14 female subjects

Results with additional simulated data:

cohort	analysis	n	n female	Median (95% CI) female vs. male	p-value (log rank)
ICI monotherapy	PFS	272	136 (50.0%)	3M (3-5) vs. 3M (3-5)	0.241
	OS	272	136 (50.0%)	10M (7-13) vs. 10M (8-14)	0.660
ICI-chemotherapy	PFS	94	47 (50.0%)	5M (3-10) vs. 6M (5-10)	0.422
	OS	94	47 (50.0%)	10M (9-NA) vs. 15M (10-NA)	0.231

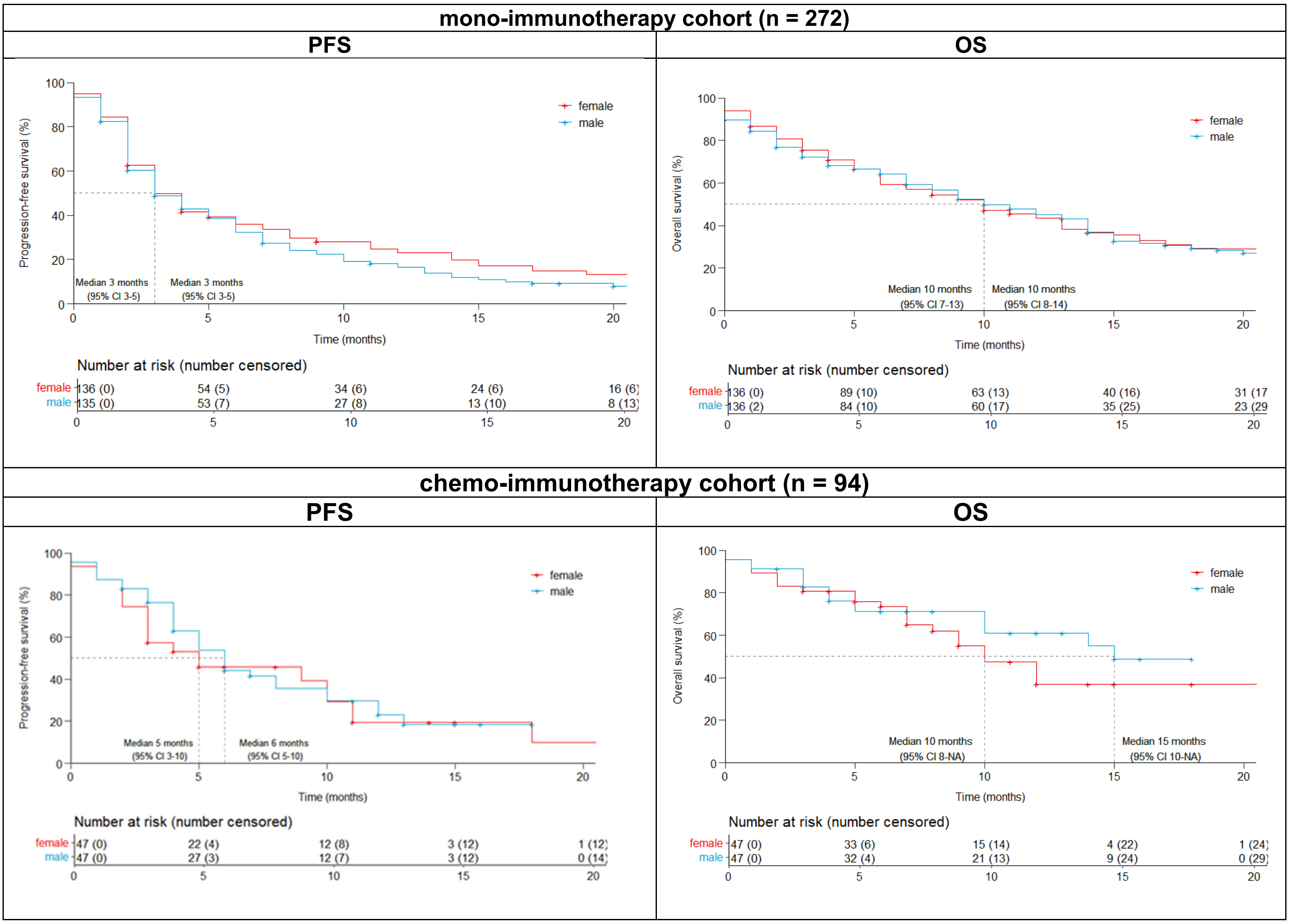

Conclusion: Simulation of a numerically balanced sex ratio in both therapy cohorts does not substantially alter PFS and OS outcomes.
